# The exon 19-deleted EGFR undergoes ubiquitylation-mediated endocytic degradation via dynamin activity-dependent and -independent mechanisms

**DOI:** 10.1186/s12964-018-0245-y

**Published:** 2018-07-05

**Authors:** Taishu Wang, Jinrui Zhang, Shanshan Wang, Xiuna Sun, Duchuang Wang, Yurou Gao, Yang Zhang, Lu Xu, Yue Wu, Yueguang Wu, Fang Liu, Xiuxiu Liu, Shuyan Liu, Yingqiu Zhang, Yang Wang, Lijuan Zou, Han Liu

**Affiliations:** 1 The Second Affiliated Hospital, Institute of Cancer Stem Cell, Dalian Medical University, Dalian, China; 2 Department of Respiratory Medicine, First Affiliated Hospital, Dalian Medical University, Dalian, China; 3 Department of Radiation Oncology, Second Affiliated Hospital, Dalian Medical University, Dalian, China; 4 Cancer Biotherapy & Translational Medicine Center of Liaoning Province, Dalian Medical University, Dalian, China

**Keywords:** NSCLC, EGFR, Exon 19 deletion, Ubiquitylation, Endocytosis

## Abstract

**Background:**

The epidermal growth factor receptor (EGFR) is closely implicated in cancer, and sequencing analyses have revealed a high mutation rate of *EGFR* in lung cancer. Recent advances have provided novel insights into the endocytic regulation of wild-type EGFR, but that of mutated EGFR remains elusive. In the present study, we aim to investigate the endocytic degradation of a frequently occurred exon 19-deleted mutant in lung cancer.

**Methods:**

The EGF-induced endocytic degradation of EGFR was examined in a panel of lung cancer cells using immunoblotting. The subcellular distribution of internalized EGFR was investigated using immunofluorescence and confocal microscopy. The effects of dynamin were assessed using its small molecule inhibitors, while the influence of RTN3 was tested using shRNA-mediated knockdown. Finally the ubiquitylation status of EGFR mutant was studied using immunoprecipitation under steady state and tyrosine kinase inhibitor-treated conditions.

**Results:**

EGF induced various rates of EGFR endocytic degradation in lung cancer cells. Interestingly, the exon 19 deletion mutant is constantly internalized and sorted to lysosome for degradation, and this process is independent of dynamin activity. EGF stimulation and HSP90 inhibition further enhance the endocytic degradation of the exon 19 deletion mutant, in a dynamin activity-dependent and -independent manner, respectively. Albeit with different modes of internalization, the uptake of the exon 19-deleted EGFR is mediated through receptor ubiquitylation.

**Conclusions:**

The internalized EGFR mutant is constantly routed through endosome to lysosome for degradation. The endocytosis of EGFR mutant occurs through both dynamin activity-dependent and -independent mechanisms. Our findings gain novel insights into the endocytic regulation of mutated EGFR and may have potential clinical implications.

## Background

The epidermal growth factor receptor (EGFR), a member of the ErbB family of receptor tyrosine kinases (RTK), plays fundamental roles during tissue development and adult homeostasis [1, 2]. The expression and activation of this RTK are tightly regulated both spatially and temporally to ensure proper propagation as well as timely termination of downstream signaling [3]. Among the multiple feedback mechanisms acquired by cells to fine tune EGFR signaling, receptor endocytosis represents a multifaceted pathway that orchestrates signal transduction and receptor downregulation [4]. Recent advances have provided compelling evidence that cells tightly control the output of EGFR activation by deploying different modes of receptor endocytosis [5, 6]. In response to various EGF concentrations, activated EGFR is internalized through clathrin-mediated endocytosis (CME) or non-clathrin endocytosis (NCE), with the former mode favors signal propagation while the latter one promotes receptor degradation to attenuate signaling. Such balance between receptor signaling and degradation is prudently maintained in normal cells but frequently decoupled in cancer cells overexpressing EGFR [7].

Accumulating evidence from cancer genomics has revealed that *EGFR* is recurrently mutated in multiple cancer types, including lung cancer, glioblastoma, head and neck squamous cell carcinoma [8, 9]. Activating mutations in EGFR renders this RTK constantly active, which in many cases behaves as a cancer driver that governs cancer growth [10, 11]. With regard to lung cancer, mutations in *EGFR* are more often detected from female, Asian, or non-smoker patients. In particular, the exon 19-deletion mutation of *EGFR* is recurrently observed in non-small cell lung cancer (NSCLC) patients, which accounts for nearly 50% of all EGFR abnormalities [10, 12, 13]. The exon 19 of *EGFR* encodes only 5 amino acids (from E746 to A750) that lie within the kinase domain of the receptor. The in-frame deletion of exon 19 confers enhanced kinase activity on mutated EGFR and thus leads to the overstimulation of downstream signaling cascades that promotes tumorigenesis.

Although the regulation of wild-type EGFR by endocytic pathways is becoming well established with recent advances and EGFR is deemed as a classic model substrate to study endocytosis, our understanding of the endocytic control of mutated EGFR remains controversial [14–19]. Impaired ubiquitylation and degradation of kinase domain mutants of EGFR were observed in lung cancer cells expressing endogenous EGFR mutants and in other cell systems with exogenous overexpression [20–23]. However, another study by Chen et al. compared a number of constitutively active EGFR mutants, and reported distinctive activation patterns, with the exon 19 deletion and L858R mutants showing increased ubiquitylation relative to wild-type EGFR upon EGF stimulation [24].

As exon 19 deletion is the most prevalent *EGFR* mutation (close to 50%) detected from non-small cell lung cancer (NSCLC) patients, the current study focused on this EGFR mutant and investigated its endocytosis [12]. Interestingly, we observed that the exon 19-deleted EGFR was constantly endocytosed and sorted to lysosome for degradation in NSCLC cells. The internalization of this deletion mutant does not require dynamin activity but relies on the ubiquitylation of RTK under steady state conditions. However, upon EGF stimulation, the exon 19-deleted EGFR was internalized through a dynamin activity-dependent mechanism. The present study thus reveals the different modes of the endocytosis of the exon 19-deleted EGFR, providing unexpected evidence towards a better understanding of the endocytic regulation of mutant EGFR. Our findings will shed light on the development of novel therapeutic strategies against NSCLC containing activating EGFR mutations.

## Methods

### Antibodies and reagents

Mouse anti-EGFR (R1), mouse anti-RTN3, mouse anti-LAMP2, rabbit anti-EEA1, and rabbit anti-EGFR (1005) antibodies were purchased from Santa Cruz. Mouse anti-Ubiquitin (P4G7) antibody was obtained from Covance. Rabbit anti-phospho-MEK1/2 (Ser217/221) and anti-phospho-AKT (Ser473) antibodies were obtained from Cell Signaling Technology. Mouse anti-GAPDH and mouse anti-β-Actin antibodies were purchased from Proteintech (Wuhan, China). Mouse anti-α-Tubulin antibody was obtained from Sigma. Goat anti-rabbit and anti-mouse IRDye secondary antibodies (infrared-labeled) were purchased from LICOR. Alexa Fluor 488-labeled and Alexa Fluor 594-labeled secondary antibodies were obtained from Invitrogen. Gefitinib, lapatinib, filipin, and dyngo-4a were purchased from Selleck. EGF was purchased from PeproTech (USA). Cycloheximide was obtained from MP Biologicals. 17-AAG was purchased from Cell Signaling Technology. Chloroquine, puromycin, and dynasore were obtained from Sigma.

### Cell culture

HEK293T and lung cancer SK-MES-1 cells were cultured in Dulbecco’s modified Eagle’s medium (DMEM) (Gibco, USA), while lung cancer cell lines A549, HCC827, H1975, H1650, H1299, and H226 were maintained in RPMI-1640 media (Gibco, USA). All cells were purchased from the American Type Culture Collection, and all media were supplemented with 10% fetal bovine serum (Gibco) and 1% antibiotics (Thermo-Fisher Scientific). All cells were grown in a humidified cell culture incubator (Thermo) at 37 °C with 5% CO_2_.

### Western blotting

Cultured cells were harvested, washed with ice-cold phosphate-buffered saline (PBS) for three times, and lysed with the RIPA buffer (10 mM Tris-HCl pH 7.5, 1% (*w*/*v*) Nonidet P-40, 150 mM NaCl, 0.1% (w/v) SDS, 1% (w/v) sodium deoxycholate) as described previously [25]. Phosphatase inhibitor cocktails (Roche) and protease inhibitor cocktails (Sigma) were added into lysis buffer immediately before use. Cell lysates were centrifuged at top speed to remove cell debris. The protein concentrations were determined using a BCA assay kit (Pierce). Each sample was mixed with the SDS-PAGE loading buffer and boiled for 5 min. Denatured protein samples were separated by SDS-PAGE, and transferred to nitrocellulose membranes. After blocking in 4% nonfat milk in PBS for 1 h at room temperature, the membranes were incubated with primary antibodies overnight at 4 °C. After incubation with secondary infrared-labeled antibodies (680 nm or 800 nm from LICOR) for 1 h at room temperature. Blots were washed 3 times with PBS and detected using a LICOR Odyssey system. Band intensity was analyzed with the Image Studio software (version 4.0) according to manufacturer’s instructions.

### Immunofluorescence and confocal microscopy

Cells were seeded onto coverslips loaded in 6-well plate and incubated overnight. After treatment, cells were washed with PBS and fixed with 4% (w/v) paraformaldehyde (Sigma) for 15 min at room temperature. Then cells were permeabilized with 0.2% Triton X100, and blocked with 10% goat serum for 30 min. Cells were stained with primary antibodies and visualized with fluorescent secondary antibodies. Fluorescence images were captured using a fluorescence microscope (Olympus BX63, Japan). Confocal images were acquired using a Leica laser scanning confocal microscope (TCS SP5).

### Immunoprecipitation

Immunoprecipitation assays were carried out as previously described [26]. In brief, treated cells were lysed with the RIPA buffer and protein concentrations were measured. One mg of protein per condition was incubated at 4 °C with anti-EGFR (R1, Santa Cruz) antibody and protein G-agarose (Roche) for 4 h. Beads were then washed 3 times with the YP-IP buffer (10 mM Tris-HCl pH 7.5, 0.1% Nonidet P-40, 150 mM NaCl), before immunoprecipitated proteins were eluted with 1.5 X SDS-PAGE loading buffer. Samples were further analyzed by immunoblotting with anti-EGFR (1005, Santa Cruz) and anti-Ubiquitin (P4G7, Covance) antibodies.

### RTN3 knockdown

Four RTN3 shRNA pGIPZ vectors (sh1 target sequence TGGATCTTCTAGATATGAA, sh2 target sequence AGGAGATGATGTTATTGAA, sh3 target sequence CCGAGATCAGACCAAGTCA, and sh4 target sequence CTCAGAAGCTTTCCATAAT) were purchased from Thermo Scientific. RTN3 shRNA lentiviruses were generated using the Thermo Scientific Open Biosystems TransLenti viral packaging system according to manufacturer’s instructions. HCC827 and H1650 cells were treated with the RTN3 shRNA lentiviruses for 24 h before treatment with 2 μg/ml of puromycin to remove uninfected cells. The efficiency of knockdown was validated by Western blottings.

### Statistics

All experiments were carried out for at least 3 times with biological repeats. Data were presented as the mean ± standard error of the mean (SEM). Statistical differences between groups were tested with two-tailed Student’s t-test using GraphPad Prism (version 5.01), with a *p*-value smaller than 0.05 considered as statistically significant.

## Results

### EGF-induced endocytic degradation of EGFR

**Fig. 1:**
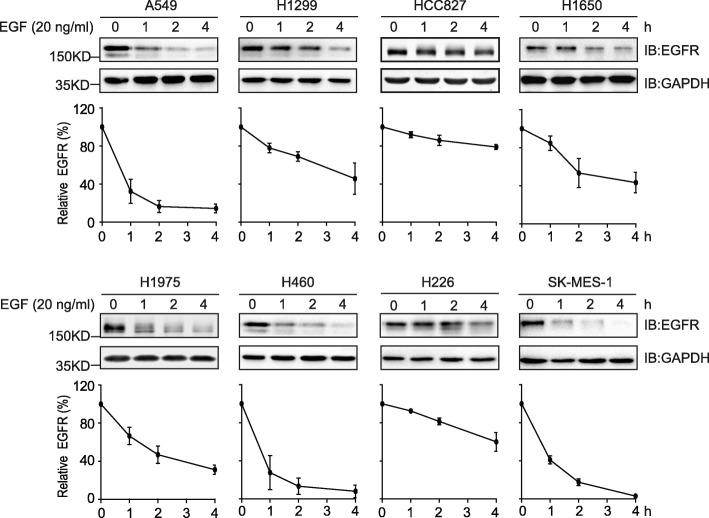
EGF treatment leads to EGFR downregulation in NSCLC cells. Indicated NSCLC cells were serum-starved and treated with EGF at 20 ng/ml for different time periods. The levels of EGFR were detected by immunoblotting assays with an EGFR antibody. The intensities of EGFR bands were quantified and adjusted according to corresponding GAPDH levels, which were used to plot degradation curves (shown below blots). All error bars represent the standard error of the mean (*n* = 3)

We first examined the EGF-induced endocytic degradation of EGFR in a panel of NSCLC cell lines, which includes all major histologic subtypes: adenocarcinoma (A549, H1299, HCC827, H1650, and H1975), large cell carcinoma (H460), and squamous cell carcinoma (H226 and SK-MES-1). HCC827 and H1650 express the exon 19 deletion mutant of EGFR; H1975 contains the T790 M and L858R double mutations in EGFR; while A549, H1299, H460, H226, and H1975 harbour the wild-type EGFR. When serum-starved cells were exposed to EGF at 20 ng/ml, downregulation of EGFR was observed in all cell types, with the fastest degradation happened in A549, H460, and SK-MES-1 cells that bear wild-type EGFR (on average to 14.3, 8, and 3%, respectively by 4 h) (Fig. 1). However, in the other two wild-type EGFR containing H1299 and H226 cells (on average to 45.7 and 60%, respectively by 4 h), the downregulation of EGFR was more slowly than that of mutant EGFR in H1650 and H1975 cells (on average to 43 and 31%, respectively by 4 h). Interestingly, the exon 19 deletion mutant of EGFR in HCC827 cells was very resistant to EGF-induced downregulation, since 4 h of EGF treatment only led to a 21% reduction of EGFR as quantified by immunoblotting densitometry. These findings suggest that, although the EGF-stimulated endocytic degradation of wild-type EGFR is rapid in certain cell types, this process of wild-type protein could also happen more slowly compared to the EGF-induced downregulation of mutated EGFR in some other NSCLC cell systems.

### The exon 19-deleted EGFR is constantly internalized

**Fig. 2:**
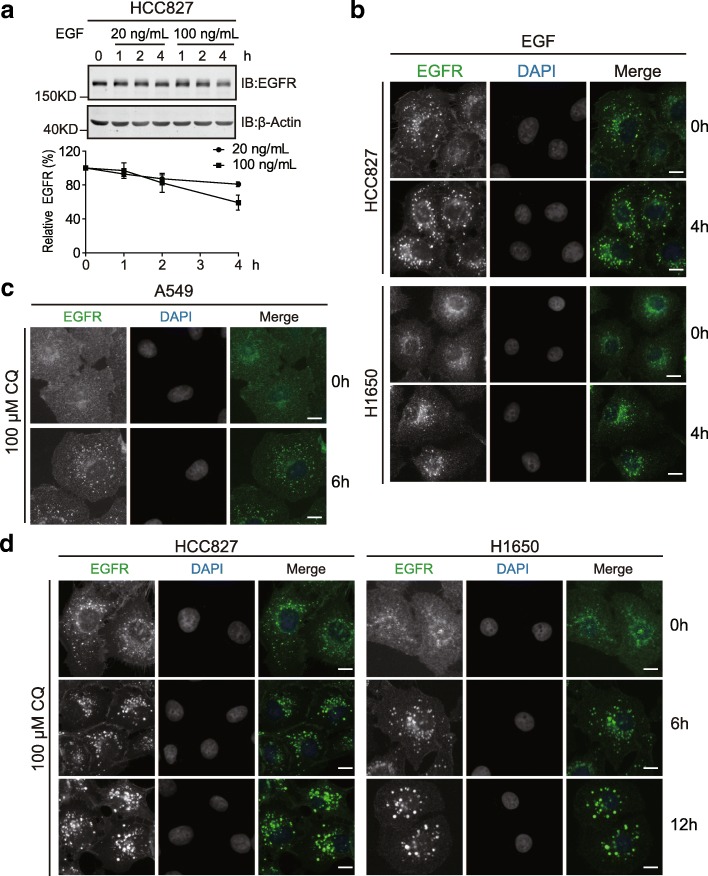
Constant endocytic degradation of the exon 19 deletion mutant of EGFR. **a**, serum-starved HCC827 cells were treated with EGF at 20 or 100 ng/ml for indicated times. Cell lysates were analyzed by immunoblottings with indicated antibodies. The graph below shows quantification data of EGFR levels. Error bars represent the standard error of the mean (n = 3). **b**, immunofluorescence experiments showing EGFR staining in HCC827 and H1650 cells with or without EGF treatment (20 ng/ml, 4 h). **c** and **d**, A549, HCC827, and H1650 cells were treated with 100 μM of chloroquine to block lysosomal degradation for indicated times before processed for immunofluorescence analysis to examine EGFR staining using a fluorescent microscope (Olympus BX63, 40X objective). DAPI stains the nucleus. Scale bar = 10 μm

We focused on the exon 19 deletion mutant of EGFR in the present study due to its prevalence in NSCLC. It is intriguing that the levels of the exon 19-deleted EGFR in HCC827 cells are very insensitive to EGF stimulation. We wondered that whether a higher dosage of EGF could enhance the downregulation of EGFR in HCC827 cells. Indeed, when HCC827 cells were treated with EGF at 100 ng/ml, the exon 19-deleted EGFR was degraded at a faster pace and on average a 41% reduction was recorded by 4 h (Fig. 2a). On immunofluorescence examination of EGFR distribution in HCC827 and H1650 cells, this RTK displayed an obvious puncta feature in the cytoplasm (Fig. 2b). EGF treatment further enhanced the formation of punctae in both cell lines harbouring the exon 19-deleted EGFR, although the increase in HCC827 cells appeared to be moderate (Fig. 2b). Hence, it seems that this mutant EGFR is more efficiently internalized than the wild-type version under steady state conditions in NSCLC cells. We then investigated whether the internalized RTK was sorted to lysosome for degradation. In wild-type EGFR containing A549 cells, blockage of lysosomal function via chloroquine led to the accumulation of EGFR in perinuclear structures, indicative of enlarged lysosomes (Fig. 2c). Similarly, in HCC827 and H1650 cells bearing the exon 19-deleted mutant, chloroquine treatment also caused a continuous increase in EGFR staining (Fig. 2d). Our observations thus revealed that both wild-type and the exon 19-deleted EGFR proteins were constitutively turned over in the lysosome.

### Dynamin activity is dispensable for the constitutive endocytosis of the exon 19 deletion mutant

**Fig. 3:**
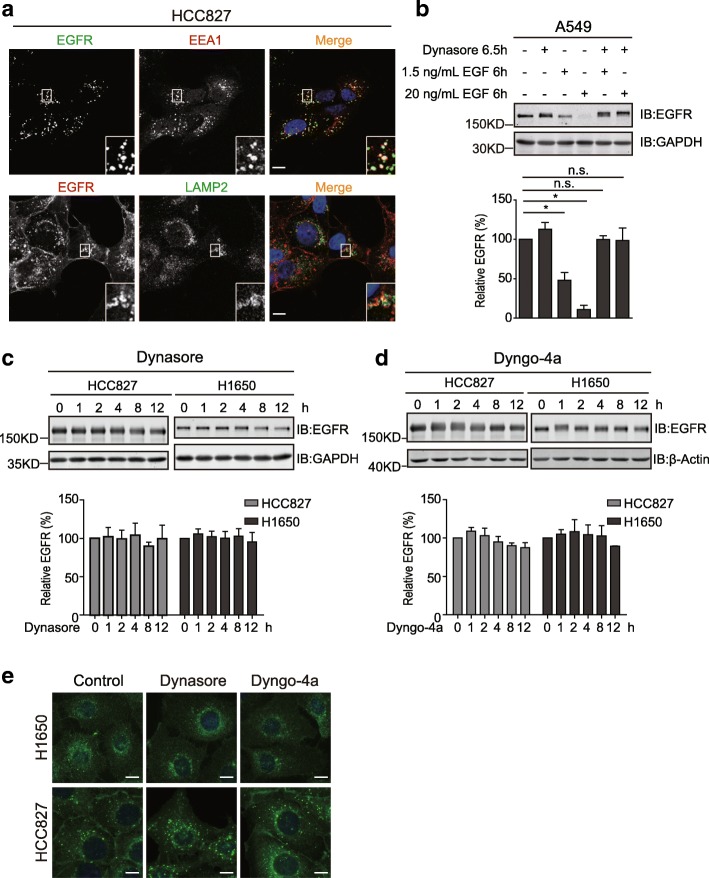
The constitutive internalization of the exon 19 deletion mutant under steady-state condition is independent of dynamin activity. **a**, representative confocal sections are shown with magnified insets to illustrate the colocalizations of EGFR with EEA1 and LAMP2 in HCC827 cells. Images were captured using a Leica TCS SP5II microscope with a 63X objective. **b**, serum-starved A549 cells were treated with EGF at indicated concentrations for 6 h with or without dynasore addition to block the dynamin activity. Cell lysates were analyzed by immunoblottings with indicated antibodies. The column chart below shows the quantification data of EGFR. **c** and **d**, HCC827 and H1650 cells were treated with the dynamin inhibitors, dynasore (80 μM) and dyngo-4a (20 μM), for indicated times. Cell lysates were subjected to immunoblotting analysis to detect the levels of EGFR. The column charts below show the quantification data of EGFR expression. **e**, immunofluorescence analysis of EGFR distribution in HCC827 and H1650 cells treated with dynasore, dyngo-4a, or control reagents. Images were captured using a fluorescent microscope (Olympus BX63, 40X objective). Scale bar = 10 μm. All error bars represent the standard error of the mean (*n* = 3), and * indicates *p* < 0.05

Having observed the constant endocytic turnover of the exon 19-deleted EGFR, we sought to investigate by which pathway this EGFR mutant was routed to lysosome. We carried out confocal microscopy experiments to examine the colocalizations of the mutant EGFR with the early endosomal marker EEA1 and the late endosomal/lysosomal marker LAMP2 under steady state conditions. As illustrated in Fig. 3a, the evident colocalizations of EGFR with EEA1 and LAMP2 reveal that, resembling wild-type EGFR, the exon 19 deletion mutant follows the classic endosome-lysosomal pathway of endocytosis.

Upon EGF exposure, wild-type EGFR is stimulated for clathrin-mediated endocytosis and non-clathrin-mediated endocytosis, both of which require the activity of dynamin [5, 27]. In support of this notion, in A549 cells carrying the wild-type EGFR, 1.5 and 20 ng/ml of EGF induced differential endocytic degradation of EGFR, while the dynamin inhibitor dynasore efficiently suppressed EGFR downregulation (Fig. 3b). Therefore, we speculated that the internalization of the exon 19 deletion mutant also required dynamin activity. Unexpectedly, the levels of the exon 19 mutant remained stable in HCC827 and H1650 cells treated with dynasore or its more potent analogue, dyngo-4a (Fig. 3c and d). In accordance with these results from immunoblottings, the subcellular distribution of mutated EGFR was also unaffected in HCC827 and H1650 cells by both dynamin inhibitors as revealed by immunofluorescence studies (Fig. 3e). These observations indicate that, although the exon 19-deleted EGFR is endocytosed through the classic endosome-lysosome pathway, its constitutive internalization occurs through a dynamin activity-independent manner under steady state condition.

### Stress-induced degradation of the exon 19-deleted EGFR

**Fig. 4:**
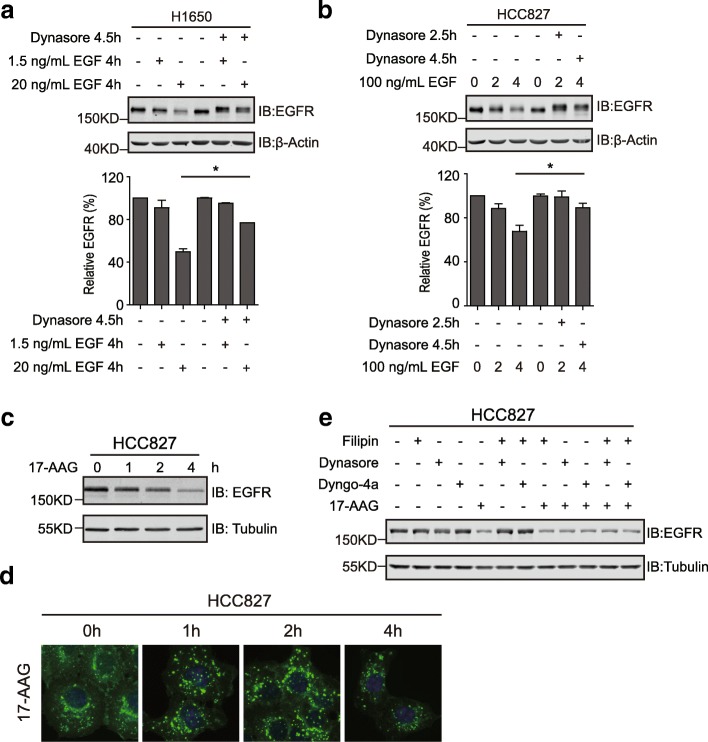
Stress-induced endocytic degradation of mutant EGFR. **a**, control and dynasore-pretreated H1650 cells were incubated with EGF at 1.5 and 20 ng/ml for 4 h, and EGFR was detected by immunoblottings. The column chart below shows the quantification data of EGFR. **b**, control and dynasore-pretreated HCC827 cells were incubated with 100 ng/ml of EGF for 2 and 4 h or left untreated (0 h). Cell lysates were analyzed by immunoblottings with indicated antibodies. The column chart below shows the quantification data of EGFR. **c**, HCC827 was treated with the HSP90 inhibitor 17-AAG for indicated times and lysed. EGFR levels were determined using immunoblottings. **d**, immunofluorescence analysis of HCC827 treated with 17-AAG for indicated times. Images were captured using a fluorescent microscope (Olympus BX63, 40X objective). **e**, HCC827 cells were treated with 17-AAG (500 nM), filipin (1 μg/ml), dynasore (80 μM), dyngo-4a (20 μM), or various combinations as indicated. Cellular EGFR levels were examined by immunoblottings. All error bars represent the standard error of the mean (n = 3), and * indicates p < 0.05

We then investigated the endocytic degradation of mutant EGFR under stress circumstances, including EGF stimulation and HSP90 inhibition. As described above (Figs. 1 and 2a), EGF efficiently induced the downregulation of the exon 19 deletion mutant of EGFR, although a higher concentration (100 ng/ml) was required in HCC827 cells. Interestingly, the dynamin inhibitor dynasore successfully blocked the endocytic degradation of EGFR incurred by EGF in both H1650 and HCC827 cells, suggesting a dynamin-dependent mechanism of EGFR internalization with EGF treatment (Fig. 4a and b).

Additionally, HSP90 inhibition has been shown to effectively trigger the degradation of mutated EGFR [28]. In line with this observation, the HSP90 inhibitor 17-AAG led to the effective degradation of the exon 19 deletion mutant in HCC827 cells (Fig. 4c). Immunofluorescence analyses revealed that EGFR was routed to lysosomes following 17-AAG treatment, suggesting that HSP90 inhibition promoted the endocytic degradation of EGFR (Fig. 4d). Nevertheless, dynamin inhibition by dynasore or dyngo-4a failed to preclude the 17-AAG-induced degradation of EGFR; and the cholesterol-interfering drug filipin that obstructs non-clathrin-mediated endocytosis did not mitigate the influence of HSP90 inhibition on EGFR levels neither (Fig. 4e). Therefore, these findings revealed the existence of multiple modes of internalization of EGFR under stress conditions.

### Reticulon 3-independent degradation of EGFR

**Fig. 5:**
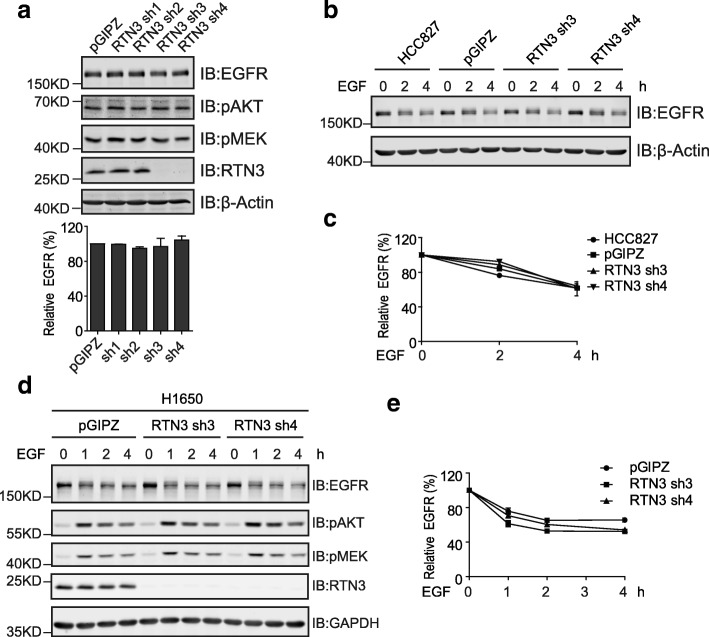
The endocytic degradation of the exon 19-deleted EGFR is independent of RTN3. **a**, HCC827 cells stably transfected with the pGIPZ control vector and RTN3 shRNA expressing vectors (sh1–4) were lysed. Cell lysates were subjected to immunoblotting analyses with indicated antibodies. The column chart below shows the quantification data of EGFR expression. **b**, HCC827 parental cells and HCC827 cells stably transfected with the pGIPZ control vector, RTN3 shRNA 3 and RTN3 shRNA 4 vectors were stimulated with EGF at 100 ng/ml for indicated times. Cell lysates were analyzed by immunoblottings with indicated antibodies. **c**, quantification data of EGFR levels in b. **d**, H1650 cells stably transfected with the pGIPZ control vector, RTN3 sh3 and sh4 expressing vectors were incubated with EGF at 100 ng/ml for indicated times. Cell lysates were analyzed by immunoblottings with indicated antibodies. **e**, quantification data of EGFR levels at indicated time points from d. All error bars represent the standard error of the mean (*n* = 3)

Recent progression on EGFR endocytosis has revealed a Reticulon 3 (RTN3)-dependent mechanism of non-clathrin-mediated endocytosis that favors EGFR degradation at high EGF concentrations [27]. To explore the involvements of RTN3 in the endocytosis of the exon 19 deletion mutant, we carried out knockdown experiments to examine the effects of RTN3 silencing on mutant EGFR. As shown in Fig. 5a, two out of four shRNAs led to excellent knockdown of RTN3 in HCC827 cells, but the levels of the exon 19-deleted EGFR and downstream pAKT and pMEK remained unaffected under steady state conditions. Further experiments investigated the endocytic degradation of EGFR stimulated by EGF treatment in RTN3-depleted HCC827 cells, and RTN3 silencing did not alter the rate of EGFR degradation (Fig. 5b and c). Similarly, in H1650 cells, RTN3 knockdown did not affect the steady state levels and EGF-stimulated downregulation of the exon 19-deleted EGFR neither (Fig. 5d and e). These observations suggest that the endocytic degradation of the exon 19 deletion mutant is exerted through a RTN3-independent mechanism.

### EGFR internalization is mediated by ubiquitylation

**Fig. 6:**
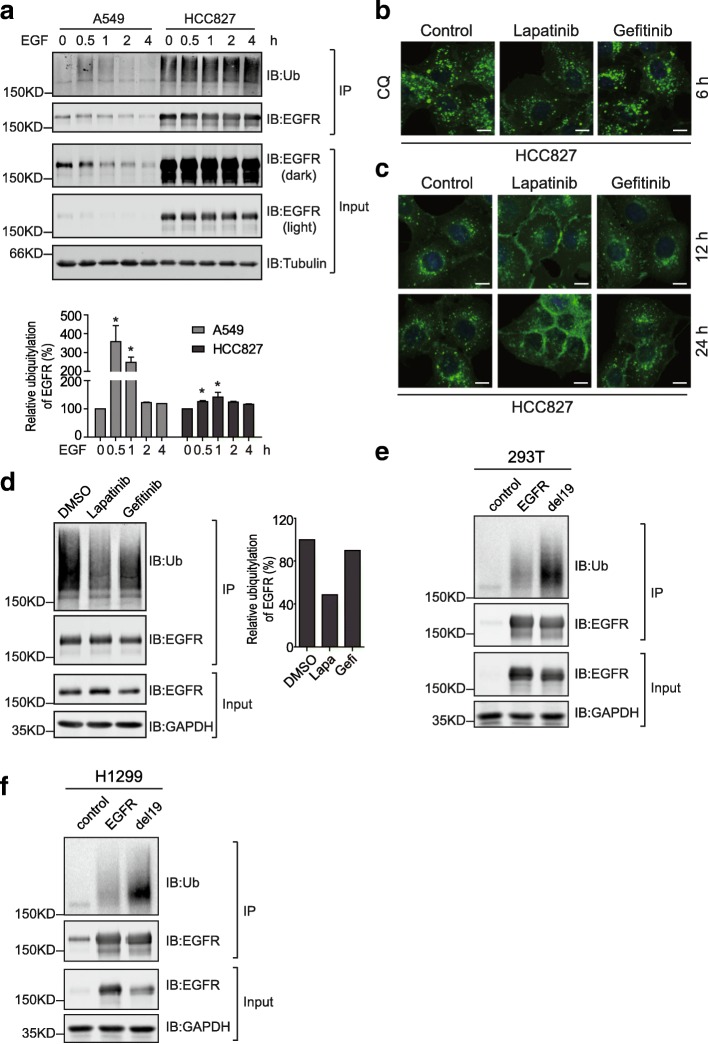
Ubiquitylation governs the endocytic degradation of mutant EGFR. **a**, serum-starved A549 and HCC827 cells were treated with EGF for indicated times. EGFR was immunoprecipitated and analyzed by immunoblottings along with input samples using indicated antibodies. The ubiquitin signal on EGFR was quantified compared to 0 h of each group and plotted. All error bars represent the standard error of the mean (*n* = 3), and * indicates *p* < 0.05. **b**, HCC827 cells were treated with lapatinib (0.5 μM), gefitinib (0.5 μM), or DMSO as control for 6 h in the presence of chloroquine (100 μM). Then EGFR distribution was examined by immunofluorescence. **c**, HCC827 cells were treated with lapatinib, gefitinib, or DMSO as control for 12 and 24 h, before processed for immunofluorescence analysis of EGFR staining. Images were captured using a fluorescent microscope (Olympus BX63, 40X objective). Scale bar = 10 μm. **d**, HCC827 cells were treated with DMSO, lapatinib, or gefitinib for 2 h and lysed. EGFR was immunoprecipitated and analyzed by immunoblottings with indicated antibodies. The column chart shows the quantification data of ubiquitin signal from immunoprecipitated EGFR. **e**, HEK293T cells were transiently transfected with control, EGFR, and exon 19 deletion mutant-expressing pCDH plasmids. EGFR proteins were immunoprecipitated and ubiquitin signal was detected by immunoblotting. **f**, EGFR proteins were immunoprecipitated from H1299 cell lines stably expressing wild-type or the exon 19-deleted EGFR before analyzed by immunoblotting with ubiquitin antibody

Considering the pivotal roles of ubiquitylation in receptor endocytosis, we examined the ubiquitylation of wild-type and mutated EGFR following EGF treatment in A549 and HCC827 cells, respectively [4, 15, 16]. As illustrated in Fig. 6a and b, the ubiquitylation signal on immunoprecipitated wild-type EGFR increases sharply at 0.5 h post EGF addition, and then declines gradually to reach basal level again after 2 h of treatment. On the contrary, we observed a strong ubiquitylation signal on immunoprecipitated exon 19-deleted EGFR from untreated HCC827 cells, while EGF addition only moderately enhanced the ubiquitylation of this EGFR mutant, although it should be pointed out that the amounts of EGFR in HCC827 is in great excess compared to A549 (Fig. 6a).

We wondered whether the kinase activity of the mutated EGFR was implicated in its endocytic degradation. Therefore, we treated HCC827 cells with two small molecule inhibitors of EGFR, lapatinib and gefitinib. Interestingly, when lysosomal function was blocked with chloroquine, the accumulated EGFR signals in cells treated with gefitinib and control reagents were significantly stronger than that in lapatinib-treated cells after 6 h of incubation (Fig. 6b). Furthermore, with prolonged treatment (12 and 24 h), lapatinib resulted in the apparent accumulation of EGFR on the plasma membrane of HCC827 cells (Fig. 6c). These immunofluorescence data indicated that lapatinib but not gefitinib reduced the endocytosis of the exon 19 deletion mutant. To investigate the different effects of lapatinib and gefitinib on EGFR endocytosis, we examined their influence on the ubiquitylation of EGFR. As described in Fig. 6d, lapatinib treatment caused a remarkable reduction in the ubiquitin signal on immunoprecipitated EGFR compared to those from control and gefitinib-treated cells. It thus seems that lapatinib disrupts the endocytosis of EGFR by decreasing its ubiquitylation.

Since the endogenous levels of wild-type and exon 19-deleted EGFR from A549 and HCC827 differed significantly, we ectopically expressed wild-type and the exon 19-deleted EGFR proteins to examine their ubiquitylation. As illustrated in Fig. 6e, HEK293T cells transiently transfected with wild-type and mutant EGFR-expressing constructs contained similar amounts of EGFR proteins, while the ubiquitin signal detected from immunoprecipitated mutant EGFR was significantly enhanced compared to that from wild-type protein. Furthermore, given that NSCLC H1299 cells expressed relatively low levels of EGFR, we established stable EGFR-expressing H1299 cell lines. In accordance with data from transient overexpression, stronger ubiquitin signal was detected on EGFR mutant immunoprecipitated from H1299 stable cells (Fig. 6f).

## Discussion

Recent years have witnessed the continuously increased mortality from lung cancer worldwide, which causes over a million of deaths annually [29]. In the United States, mortality from lung cancer accounts for more than 25% of cancer-related deaths [30, 31]. Therefore, the demand for effective therapies against lung cancer is huge, and many efforts have been made to develop novel therapeutic strategies. Accumulating evidence from sequencing analyses has revealed the high frequency of *EGFR* mutations occurring in lung cancer, among which the exon 19 deletion appears to be the most prevalent one. Compared to wild-type EGFR, the exon 19 deletion renders mutated EGFR constantly active, and therefore overactivates a wide array of signaling pathways. Nonetheless, this EGFR mutant exhibited sensitivity to multiple tyrosine kinase inhibitors. EGFR-targeted therapy represents one of the most successful approaches in the treatment of lung cancer, which has been approved by the FDA as first-line therapy that effectively prolonged patient survival [32, 33]. However, cure is seldom achieved, and relapsed disease acquires drug resistance to threaten patient lives. The most prevalent mechanism of developed resistance to EGFR inhibitors, such as gefitinib and erlotinib, is attributed to secondary mutations occurring in *EGFR* [34, 35]. Considering the critical roles of EGFR in lung cancer tumorigenesis and drug resistance, the thorough investigation of its features in lung cancer is prerequisite for the development of novel therapeutic strategies in the treatment of lung cancer.

In the present study, we carefully examined the EGF-induced degradation of endogenous EGFR proteins in a panel of non-small cell lung cancer cell lines. Through comparing the degradation rate of wild-type and mutated EGFR in cells belong to different histologic types, we drew the conclusion that being wild-type or mutated form of EGFR alone could not determine the degradation rate upon EGF treatment; rather, the EGF-stimulated downregulation of EGFR is controlled by multiple factors that differ within various cellular contexts [3, 15, 16]. With a closer examination of the exon 19 deletion mutant of EGFR in HCC827 and H1650 cells, our observations reveal that this mutant undergoes endocytic degradation constantly. Under steady state conditions, the exon 19-deleted EGFR forms a number of intracellular punctae that colocalize with endocytic compartments; while the amounts of intracellular EGFR punctae reversely correlate with the efficiency of EGF-induced EGFR downregulation. In HCC827 where a large amount of EGFR punctae exist, the exon 19 deletion mutant appeared to be the least sensitive to EGF-induced endocytic degradation. A possible explanation to this phenomenon is that the mutated EGFR already occupies the endocytic routes to lysosome, thus causing a ‘traffic jam’ that limits the capacity of further transport; while the situation is even more severe in HCC827 cells due to a large surplus of EGFR. These features collectively led to the inefficient EGF-induced endocytic degradation of EGFR in HCC827 cells.

In our attempt to investigate the molecular determinants of mutant EGFR endocytosis, we observed dynamin activity-dependent and -independent mechanisms. Since the EGF-induced endocytosis of wild-type EGFR has been well-documented, a simple assumption is that mutated EGFR follows similar mechanisms. Initially, we speculated that the endocytosis of the exon 19 deletion mutant might primarily adopt the non-clathrin mediated pathway, resembling that of wild-type EGFR under high EGF conditions, considering that the mutation rendered EGFR constantly active. Nevertheless, under steady state conditions, the internalization of the exon 19 mutant is independent of RTN3 and dynamin activity that are involved in the non-clathrin mediated endocytosis [27]. The dynamin activity is also dispensable for the enhanced endocytic degradation of EGFR elicited by HSP90 inhibition. Dynamin is nonetheless implicated in the EGF-stimulated endocytosis of the mutated EGFR. These findings reveal that both dynamin activity-dependent and -independent mechanisms function in the endocytic regulation of the exon 19 deletion mutant.

**Fig. 7:**
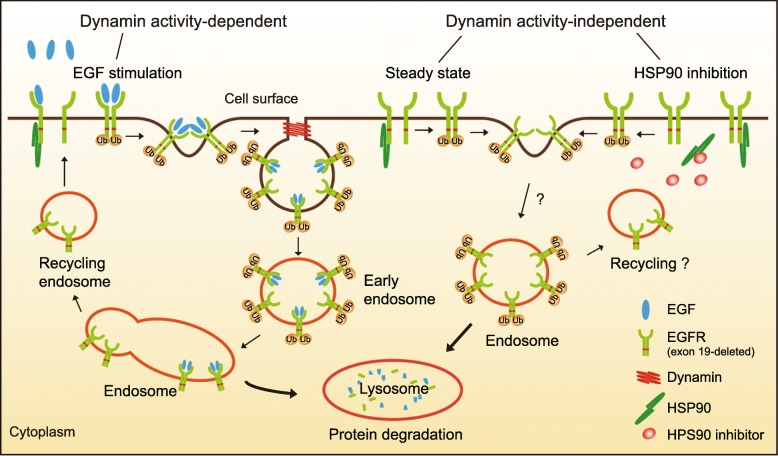
A working model depicting the different modes of endocytic regulation of the exon 19 deletion mutant. Both dynamin activity-dependent and -independent mechanisms are implicated in the endocytic regulation of the exon 19-deleted EGFR in NSCLC cells. With EGF stimulation, the mutant EGFR is internalized through a dynamin-dependent pathway. However, under steady state and HSP90 inhibition conditions, the dynamin activity is dispensable for the uptake of the EGFR mutant. The internalized receptor travels through the canonical endosome-lysosome route, during which process receptor ubiquitylation plays a pivotal role albeit of various modes of endocytosis

A series of studies on the endocytic regulation of wild-type EGFR have provided compelling evidence that this RTK is internalized through different dynamin-dependent pathways in response to low and high EGF concentrations [5–7, 27]. It is noteworthy that, when both dynamin-dependent pathways were disrupted, a small fraction of endocytosis (10–15%) of wild-type EGFR was still observed, suggesting the possible existence of alternative means of EGFR internalization [5]. Therefore, we speculate that the dynamin activity-independent pathway(s) may be implicated in the endocytosis of the exon 19-deleted EGFR under steady state and HSP90-inhibited conditions (Fig. 7).

Further investigation confirmed the evident ubiquitylation of the exon 19 deletion mutant under normal conditions, which was less sensitive to EGF stimulation but could account for the constant endocytosis of the receptor, considering the dominant functions of ubiquitylation in receptor endocytosis. Through manipulating the ubiquitylation levels of the mutated EGFR with small molecule tyrosine kinase inhibitors (lapatinib and gefitinib), we observed accumulated cell surface distribution of the receptor in lapatinib-treated cells, indicative of impaired endocytosis with correlated reduced ubiquitylation of EGFR. Although lapatinib and gefitinib are both quinazoline derivatives, they show different binding preference towards EGFR [36]. Lapatinib binds to the inactive conformation of EGFR and prevents its dimerization; while gefitinib binds to its active kinase domain and promoted the formation of dimers, which explain their different effects on receptor ubiquitylation [37]. These findings emphasize the pivotal roles of ubiquitylation in receptor endocytosis, which governs receptor endocytosis with different pathways of choice.

## Conclusions

To summarize, our observations revealed that the exon 19 deletion mutant of EGFR is constantly internalized and routed through endosome to lysosome for degradation in lung cancer cells. Interestingly, the endocytosis of EGFR mutant involved both dynamin activity-dependent and -independent mechanisms, and differed from that of wild-type receptor. Importantly, receptor ubiquitylation appeared to play a pivotal role in the endocytic degradation of EGFR. Therefore, further investigation is warranted to tease out the precise mechanisms of the internalization of mutated EGFR, which will assist the development of novel EGFR-targeted therapeutic strategy in the treatment of lung cancer.

## Abbreviations

CME: Clathrin-mediated endocytosis
DMEM: Dulbecco’s Modified Eagle’s Medium
EGFR: Epidermal growth factor receptor
NCE: Non-clathrin endocytosis
NSCLC: Non-small cell lung cancer
PBS: Phosphate-buffered saline
RTK: Receptor tyrosine kinase
RTN3: Reticulon 3
SEM: Standard error of the mean
